# Supplementary figures and images for: Bat Rabies in the Americas: Is Myotis the Main Ancestral Spreader?
Source: Viruses. 2024 Aug 16;16(8):1302. doi: 10.3390/v16081302 (PMC11359690; doi:10.3390/v16081302)

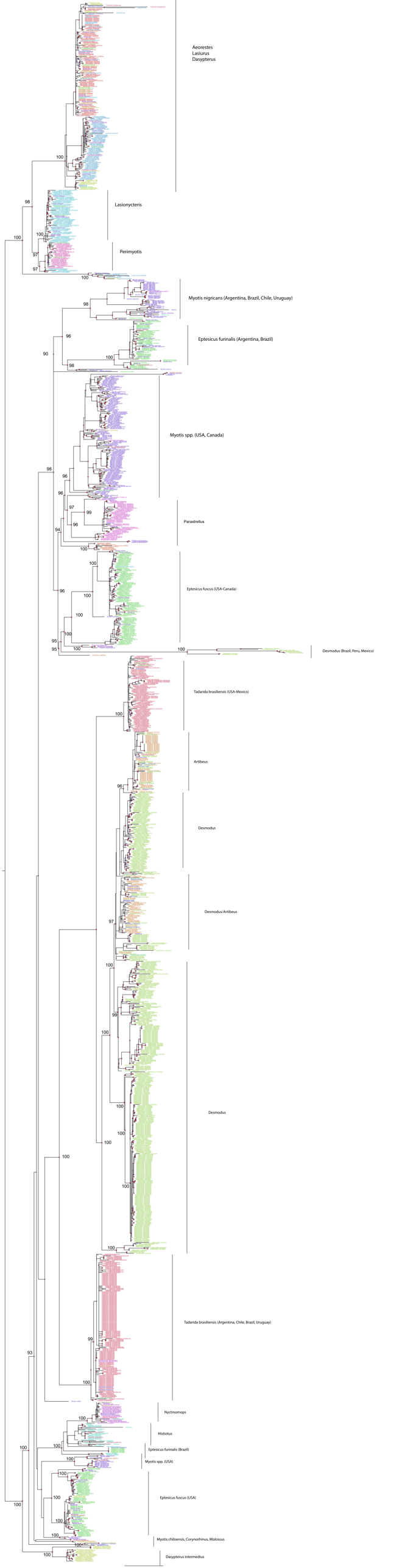

Supplement: Supplementary file 1 [file viruses-16-01302-s001.zip › S1 Fig.pdf]
